# Principles of Medicine: Comprising General Pathology and Therapeutics, and a Brief General View of Etiology, Nosology, Semeiology, Diagnosis and Prognosis

**Published:** 1844-02

**Authors:** 


ART VI.—*Principles of Medicine: comprising general Pathology and Therapeutics, and a brief general view of Etiology, Nosology, Semeiology, Diagnosis, and Prognosis.* By CHAS. J. B. WILLIAMS, M.D., F.R.S., Fellow of the Royal College of Physicians; Professor of the Principles and Practice of Medicine, and of Clinical Medicine, and First Physician to the Hospital, University College, London; Consulting Physician to the Hospital for Consumption and Diseases of the Chest, &c. *With Additions and Notes:* By MEREDITH CLYMER, M.D., Lecturer on the Institutes of Medicine; Physician to the Philadelphia Hospital; Fellow of the College of Physicians, &c. &c. Philadelphia: Lea & Blanchard. 1844. 8vo. pp. 383.

This work supplies a deficiency long felt by the profession —the want of an elementary treatise on General Pathology. The author of it has had extensive experience at the bed-side, and has drawn chiefly from his own observation of disease as he has witnessed it in hospitals and private practice, during upwards of twenty years. The want of this union of science and practice in the same individual, he regards as the cause of the admitted fact, that practical medicine has not kept pace with the rapid improvements in the contributory sciences. He adds, we fear, with too much truth, "that scientific men are not and cannot be practical, because they have had no experience; and practitioners know little of science, and therefore derive little good from it. Instead of working together, these parties are at issue with each other. But it is high time," he continues, "to put an end to this feud. Philosophers must descend from their transcendental positions, to consider the details of practice and purposes of utility."

Without further preface, we proceed at once to notice a work which proposes thus to combine practice and science, and as our notice is designed to be analytical we shall not stop to remark on the introductory chapter, which insists with great force on "the need of principles in medicine."

Chapter I is devoted to the causes of disease, and the first observation is, that—

"Disease sometimes originates within the body from a change in some of the component parts of the animal frame, without any obvious external influence; thus disorder may arise from an undue proportion or predominance of a function, as that of the nervous system; or of a constituent of the body, as in the case of sanguineous plethora. Such states, however, constitute more commonly proclivity to disease than disease itself; and generally disease arises from causes extraneous to the body, although in many instances we may fail to detect these causes."

He adopts the division of the causes of disease, long sanctioned by the profession, into predisposing and exciting, the most numerous of the former of which he remarks are debilitating causes. Thus low fevers, and epidemic and contagious disorders, are rapidly propagated among an ill-fed population. Happily, we have yet but little experience of this in our highly-favored country. But of the truth of the proposition no one can entertain a doubt. The other predisposing causes are as follows: Confinement in impure air, excessive exertion of mind or body without sufficient sleep, long continued heat, long continued cold, habitual intemperance with intoxicating liquors, distressing passions of the mind, excessive and repeated evacuations, either of the blood or of some secretion from it, previous debilitating diseases, and the treatment used to relieve them. Hereditary conformation, and age, are also referred to as predisposing causes.

The exciting causes are next enumerated. He divides them into two classes; to-wit, cognizable agents, and such as are not cognizable. The first class embraces mechanical and chemical agents, ingesta, bodily exertion, mental emotion, suppressed or defective evacuations, excessive evacuations, temperature and changes. In the second class are cited endemic, epidemic and infectious poisons. We deem it sufficient to announce the subjects. The reader will supply the matter under each head from his previous study or observation.

Chapter II treats of *pathology proper*, and in the several sections are discussed diseased irritability, excess of tonicity, diseases of sensibility, diseases of voluntary motion, diseases of sympathetic nervous influence, and diseases of secretion. Under the head of sympathetic nervous influence, he refers to the following pathological phenomena:

"Clinical observation teaches us that not merely motory impressions, but those also which cause sensations, may be reflected, so that when the impression is made on one part, the sensation is experienced in another. I do not allude to the fact that a stroke on the nervous trunk produces feelings referred to its branches, but I advert to impressions on the ultimate distribution of one nerve producing sensations in parts supplied by another nerve, or by another branch of the same nerve. The following are examples of this kind. Touching the external auditory meatus causes a tickling sensation in the glottis. A calculus in the bladder produces pain referred to the extremity of the penis. Ascarides in the rectum cause itching of the anus, and sometimes of the pudenda. Congestion of the liver sometimes is accompanied by pain in the right shoulder-blade; and a disordered state of the stomach, occasionally with pain in the left shoulder-blade. The pains of angina and gastrodynia often extend to the whole chest, and the former especially radiates to the left arm. Severe frontal headache is almost instantly caused in some persons by acid ingesta, in others by eating ice. Irritation of the intestines, as in cholera and colic, (especially painter's colic), frequently causes pain and tenderness in the legs and feet, even when there has been no cramp or other excitomotory phenomena. Temporary neuralgic affections seem to be due to similar causes."

*Diseases of the constituents of the blood* are described under the head of diseased secretion. We quote extensively from this part of Dr. Williams' work, as many of the details are new and curious. The constituents of the blood, to be considered as in excess, defect, and alteration, are the red globules, the fibrine, albumen, oil, salts, and water. The average natural proportions of the chief constituents of the blood, according to the best analyses, are 127 red globules, 3 fibrine, 72 animal matter in the serum, 8 salts, and 790 water.

*Changes in the Red Particles.*—"Excess of the red particles might therefore be expected to cause a general excitement of the vital properties of the body. Accordingly, Lecanu found that they exist in larger proportion in persons of sanguine temperament than in others, and more in males than in females. Andral and Gavarret detected an excess in the early stage of inflammation and fevers, especially eruptive fevers, as measles and scarlatina. In sanguineous plethora, also, and in hæmorrhagic diseases before much blood is lost, they were in excess, in some instances rising to 185 in 1000 of blood. The obvious sign of the abundance of red particles is the florid color apparent in the lips, cheeks, gums, and other vascular parts; the deep-blue color of the superficial veins; and the fine deep crimson which a thin film of blood gives on a white plate. The functions, animal heat, and muscular irritability are in an elevated state, bordering on or passing into febrile excitement."

"The *red particles are defective* in persons of the lymphatic or leucophlegmatic temperament; after great losses of blood, (artificial or from disease); in chlorosis, and in other anæmic states, as those connected with advanced stages of cancer, diabetes, scurvy, and other cachectic diseases; in scrofulous and tuberculous diseases; in the latter periods of fevers, and after severe inflammations; in granular degeneration of the kidney, and other organic diseases attended with dropsy; in diseases of the spleen, and others of malarious origin. In extreme cases of chlorosis, the proportion of the red particles was found, by Andral, reduced to 28 in 1000 of blood.

"The signs of the defect are, paleness of parts naturally colored with blood, pallid or sallow hue of the skin, pink color of superficial veins, and a pinkish or light purplish hue of a film of blood on a white plate. The symptoms of such a condition are those which will be more fully described under the head of anæmia; a weak state of the functions generally, of circulation, calorification, digestion, and nutrition, constituting their summary."

"The *red particles* are evidently *altered* in some diseases, the coloring matter being much darker than usual, as in the worst forms of scurvy, in which the blood is said, by Mead, to be changed to a dark brown or green color: in the Walcheren and other malignant fevers it has been described as pitchy black. Some change seems to occur in congestive typhoid fevers, in which the blood-vessels become stained or dyed of a deep claret color; this imbibition implies an unnatural solution of the red particles. Probably the occurrence of petechiæ and ecchymosed patches in these diseases is partly dependent on a similar change. The readiness with which the textures become stained in scorbutic subjects, and in secondary syphilis, seems to indicate an alteration in the coloring matter; all inflammations and ecchymoses in the skin being followed by livid, purple, or copper-colored stains. The black matter of melanosis seems to be the coloring part of the blood in an altered state: this is certainly true of the spurious melanosis of the intestines."

"*Remedial Agents.*—Excess of the red particles may be speedily removed by blood-letting, which reduces these much more than the other constituents of the blood. Low or vegetable diet, and the antiphlogistic regimen generally, produce a similar effect more tardily. It is not certain whether any medicines directly act in a similar way; but probably the continued use of mercury, colchicum, and other medicines which largely increase the excretions, ultimately reduce this element. The remarkable pallidity which accompanies the occurrence of extensive suppuration would also point to the formation of pus as a means of diminishing the red particles, which means may be used artifically in the form of setons and suppurating counter-irritants.

"To *promote the increase* of the red particles, *where defective*, we might expect nourishing food, especially meat, exposure to invigorating air and light, with tonics generally, to be the proper means. But without experience we could not have anticipated that medicines containing iron should possess such remarkable efficacy in relation to this element of disease. In many cases of chlorosis, under the use of any suitable preparation of iron, the complexion will change from waxy to ruddy, in three or four weeks' time. This subject will again come under our consideration in connection with anæmia."

*Fibrine.*—"An *excess of fibrine*, and of the colorless or lymph-globules, exists in inflammatory diseases, especially those of a sthenic character, and acute rheumatism. In some cases, MM. Andral and Gavarret found it as high as ten per thousand. The proportion of fibrine is also increased during the latter months of pregnancy. These facts have been long known; but in addition to these MM. Andral and Gavarret found an excess of fibrine in tuberculous diseases, in which we have noticed there is a defect of red particles. Mr. Gulliver has observed the increase of white globules in blood drawn in inflammation; and I have noticed this as occurring in the vessels."

"*Deficiency of fibrine* is of frequent occurrence in many diseases and temporary conditions bordering on disease. Its sign is fluidity or imperfect coagulation of the blood when drawn. As venous blood contains less fibrine than arterial, so the quantity is absolutely diminished when the blood is more venous than usual, as in cases of asphyxia or impeded breathing; and in those of cyanosis, in which the venous blood becomes mixed with the arterial through an unnatural opening. Excessive bodily fatigue and want of sleep expend the fibrine: hence the blood remains fluid in animals hunted to death. It was stated by John Hunter that the same thing is observed in animals killed by lightning; but this is not always the case. In many instances the blood is found fluid in cases of death from poisoning and other sudden causes. In some of these the absence of fibrine may be attributed to the impeded respiration which is the immediate cause of death, as in some cases of death from hydrocyanic acid, opium, strychnia, apoplexy, dividing the pneumo-gastric, (Dupuy), &c. There is, however, some uncertainty about these facts. (See Mr. Blake's experiments mentioned further on). But in others, as in poisoning with arsenic, sulphuretted hydrogen, and some other pernicious agents, the fluid state of the blood must be ascribed to a more direct operation on the blood itself. So likewise in adynamic fevers, which arise from a peculiar poison, the fluidity or imperfect coagulation of the blood is one of the most remarkable conditions, and seems to be a chief cause of the hæmorrhages, petechiæ, and vibices, which sometimes occur in these fevers. In a case of very low typhoid fever, Andral found the proportion below one in one thousand. The artificial imitations of these fevers produced in dogs inoculated with various morbid or putrid matters, or confined over their exhalations, in the experiments of Gaspard, Majendie, Gendrin, Leuret, and Hamon, exhibited a similar absence of fibrine in the blood."

"*Alterations in the quality of the fibrine* introduce to our notice the important morbid appearances presented by the buffy coat and contraction of the clot of blood.

"As the consolidation of the fibrine is the cause of the coagulation of the blood, so differences in the coagulum represent variations in the properties of the fibrine.

"A large firm coagulum indicates an abundance of fibrine, as well as of red particles, and is commonly presented by healthy blood. A loose coagulum implies a deficiency of fibrine. A small firm clot betokens a proportion of fibrine exceeding that of the red particles; but the smallness of the clot points to another property of fibrine, which is of excess, during and after its consolidation. Again: in this case as in others, the upper part of the clot is commonly more contracted than the lower portion; it is also firmer and contains more fibrine, whilst the lower abounds more in red particles. Here there is evidently a tendency to a separation of the red particles from the fibrine. In other cases, again, the separation is to some extent complete, the red particles subsiding, whilst the fibrine rises to the surface and on coagulating forms at the top of the clot a layer of a light yellow or buff color, commonly known by the name of the *buffy coat.*"

"*Remedial Agents.—Excess* of fibrine is less directly reduced by blood-letting and low diet, than is excess of the red particles; yet these are the chief means of lowering the quantity of fibrine in the blood. It would probably be found that purgatives and other remedies which increase much the more solid secretions, diminish the fibrine. A similar property has been ascribed to mercury, to alkaline salts, to iodine, and to antimony. I know of no positive facts in support of this notion; but it is favored by some analogies, and seems well worthy of experimental investigation. The operation of salts and alkalies in this way was probably suggested by their property of dissolving fibrine out of the body.

"According to the views of Liebig, subsisting chiefly on saccharine, amylaceous, or gelatinous articles of food, must reduce the fibrine and albumen of the blood; and such food is found by experience to be the best in inflammatory diseases, in which excess of fibrine is a chief element. Is the reputed efficacy of the 'cure de raisains,' in tuberculous disease, connected with the absence of protein compounds in the food? Bodily exercise reduces the fibrine, and may be advantageously employed with this view in sthenic plethora; but is not admissible in inflammatory diseases. Neither can we suggest any practicable mode of lessening the fibrine by lowering the function of respiration, on which its supply seems to depend, unless narcotics, which impair many organic functions, have some action of this kind. The known utility of opium, aconite, &c., in rheumatism and low forms of inflammation, in which excess of fibrine is a constant element, makes this matter deserving of some research.

"*Deficiency of fibrine* is to be remedied by assisting those functions on which its supply depends, particularly those of digestion, respiration, and assimilation, and by avoiding its expenditure in too much exercise and other exhausting processes. If the digestive organs will bear them, meat, eggs, bread, and other articles of diet abounding in the protein compounds, should be taken. The digestive and assimilative functions may be assisted by stimulants, bitters, quinine, and the mineral acids, which from their power in stopping passive hæmorrhage, and in augmenting the muscular strength, seem to promote the formation of fibrine more directly than by their mere operation on the digestive organs. To improve the function of respiration, besides attempts to remove or diminish any disease from which it may suffer, the free access of pure cool air to the lungs should be secured. The injurious effect of exertion is exemplified in the relapses which it often induces in continued fever, in which defect of fibrine in the blood is a chief element. Fatigue of every kind, and wakefulness, should be carefully avoided, and sleep obtained by narcotics, if it do not come naturally. In case of any deficiency of fibrine from the presence of a febriferous or putrescent poison in the system, it is not to be expected that fibrinous food, rest, or any other means, can remove the deficiency, so long as the poison remains in active operation. This poison, by its septic or analogous influence, interferes with the vital process by which the fibrine is formed. But no sooner does the influence of the poison subside, as evidenced by improvement in the symptoms, than the quantity of fibrine increases; and this sooner than could be explained by any increase of nourishment taken. (Andral and Gavarret)."

"*Excess of albumen* exists in most cases of inflammations and fevers, especially during their more active stages. Its increase is not, however, in proportion to that of fibrine. Its relative proportion is much increased in epidemic cholera; but this is rather due to the removal of the water of the blood. Albumen is the principle least affected in its proportions by disease. Very poor living, long continued, extensive hæmorrhages, and other drains on the system, will pretty surely reduce it in common with the other animal principles of the blood; but good living has less power in raising it above the natural standard.

"*Deficiency of albumen* in the blood is most remarkably met with in cases of albuminuria, or diseases of the kidney with coagulable urine; and this deficiency precedes the diminution of the red particles, which takes place in the advanced stages of this disease. Dr. Bright found in a patient with albuminuria, the specific gravity of the serum as low as 1013. Dr. Babington found the specific gravity of the serum in a case of diabetes as low as 1024; in another 1027, although that of the blood was higher than usual, 1061. In this case the serum was milky. In their latter researches, MM. Andral, Gavarret, and Delafond discovered a remarkable diminution of the albumen in dropsical sheep affected with the rot, (a water state of the blood, with distoma in the liver). Sheep in a cachectic state, with a deficiency of red particles, but without entozoa, were not dropsical, and in these the albumen was found undiminished. It is therefore most probable that the cases of cachexia, or anæmia, attended by dropsy, owe this concomitant to a defect of albumen in the blood. It is this principle chiefly that gives the blood liquor its spissitude, which renders it more fit to pass along the vessels, and prevents it from transuding through their walls. This deficiency of albumen, therefore, seems to be a chief constituent of the diathesis."

The increase of fat in the textures is preceded, Dr. Williams supposes, by its presence in excess in the blood, and is favored by a diet composed of oleaginous, saccharine and farinaceous substances. Exercise counteracts the tendency to it, "probably by causing the combustion of the fat in respiration, whilst muscular textures are increased by the same influence."

The salts of the blood are diminished in many diseases, but it is not known that they are augmented by any. Their deficiency has been particularly remarked in malignant cholera, to which circumstance the temporary efficacy of injection of saline solutions into the veins of the cholera patients may be fairly attributed. Some pathologists have stated that a similar change in the blood occurs in typhus and yellow fever, but this assertion seems not to have been supported by the researches of Andral.

The proportion of water in the blood increases with the diminution of the animal constituents. It is thus found in excess after extensive hemorrhages, and in chlorosis, and other states of the system marked by anæmia. Andral cites the case of a woman in whom the blood, after repeated menorrhagias, contained, in red globules, only 21 parts; in fibrine 18; in solid matters of the serum 61; the water being 915 in 1000. The tendency is to dropsy, in such a condition of the circulating fluids. The opposite state of the blood exists in cholera, in which this fluid becomes so thick that it finally circulates with difficulty, as is evinced by the lividity, feeble pulse, and other signs of obstructed circulation.

Under the head of *changes in the blood by respiration*, we meet with the following instructive observations:

"In cases of cyanosis, (the blue disease, in which, from malformation of the heart, some venous blood passes into the arteries), we have the opportunity of observing the more essential effects of defective arterialization of the blood. Individuals thus affected are in a lower scale of animation. The slower processes of nutrition and secretion seem to go on pretty well, but the muscular power is low; slight exertions bring on symptoms of faintness, palpitation, suffocation, or insensibility; the animal heat is lower than natural, and there is greater suffering from the influence of cold. In short, all the powers of body and mind are slender, and are easily disordered by any circumstances which tax their activity. In the few that reach mature age, there is no sexual passion, which seems to be a happy provision against the chance of perpetuating a malformed race—human reptiles. The subjects of cyanosis are said to be very liable to hæmorrhages, and when these occur spontaneously, or from accidental causes, it is very difficult to stop them. This must be ascribed to a defect of fibrine which we have already found to occur where the changes of the blood by respiration are imperfect. The same defect occurs in the fœtus.

"In connection with the scantiness of fibrine in the blood, when the respiratory changes are defective, we must notice the weakness of the muscles generally, which are probably nourished by the fibrine. This weakness is often observed in the subjects of extensive disease of the lungs, especially emphysema. In these same subjects the deposition of fat is, on the other hand, often excessive, which agrees very well with Liebig's idea that respiration directly consumes the oily parts of the blood; the respiration being defective, the fat accumulates."

Important changes in the blood are effected by the secretions, of which a very striking instance is afforded by defective action of the kidneys. These organs having been extirpated, the animals subjected to the operation died in a few days, affording after death evident traces of urea in the blood. Defective secretion of bile must be attended by many serious evils, but the effects have not been so accurately determined. Our author believes that some connexion exists between this state of the liver, and purpura, in several cases of which disease he noted a want of biliary secretion. The function of the skin when impaired is apt to be followed by rheumatism, urinary disorders, and cutaneous diseases. The reason assigned for this by Dr. W. will shock the opposers of the humoral pathology. He ascribes it all to the retention in the blood of the lactic acid and lactates of soda and ammonia, proceeding from the transformation or decay of the textures, along with the perspirable matter. "Hence," he says, "the remedies for rheumatism should not be merely antiphlogistic, but also of a kind calculated to eliminate the morbid matter from the blood."

In chapter III we have an account of "*Secondary or proximate elements of disease consisting of two or more primary elements.*" *Anæmia* is one of these—a condition, the peculiarity of which is a deficiency of blood. It is often symptomatic, but sometimes arises without any other known disease. Dr. Clymer says:

"The fundamental and constant character of anæmia is the diminution in the red corpuscles of the blood. Women are more subject to it than men, although these may be attacked with spontaneous anæmia at all ages. The clot is small, but firm and dense, swims in a large quantity of colorless serum, and presents on its surface a well-marked buffy coat, which, however, differs from the buff of inflammatory blood by a gradual termination in the red-mass, and not by an abrupt, well-defined line."

*Hyperæmia*, the blood in excess, is a most frequent element of disease. It embraces general plethora, congestion, and inflammation, with the results—hemorrhage, flux, dropsy, &c. It will be impossible, in the limits assigned us, to give an analysis of the interesting matter presented under each of these heads. We shall be compelled, therefore, to select a few of the most practical portions.

On the treatment of congestions, the following judicious remarks are made:

"The operation of several of the foregoing agents, in combination or succession, is generally more effectual than that of single ones in the cure of congestions. Thus congestion of the liver may resist the action of mercury, and may even be aggravated by it, until the vascular distention has been partially reduced by local blood-letting or derivants; then the mercury, by increasing the secretion, reduces the remaining congestion. Congestion of the kidneys is augmented rather than diminished by diuretics, which then fail to increase the secretion of urine, but may only render it more albuminous. But after some relief has been given by cupping to the loins, and hydragogue purgatives and diaphoretics, then some diuretics, particularly digitalis and cantharides, cause a freer flow of urine, with less albumen. The same point might be further exemplified; but it is unnecessary to multiply instances."

Hemorrhages constitute a most embarrassing class of diseases. Nothing beyond the outlines of treatment can be laid down in a work like this. They are thus given:

"We have now to consider the means calculated to restrain all kinds of hæmorrhage, and which is especially opposed to the causes which more immediately determine this result of disordered circulation. If blood-vessels are softened, brittle, or actually ruptured or ulcerated, a chief thing to be done is to diminish the quantity of blood sent to them; and, besides by blood-letting, this may be effected by pressure, posture, cold and astringent applications, and means calculated to tranquilize the whole circulation. Thus epistaxis is sometimes arrested by pressure on the carotids; uterine hæmorrhage by pressure on the abdominal aorta, or by elevating the pelvis; hæmoptysis by keeping the chest high; and in all cases of hæmorrhage, perfect stillness and a cool regimen should be observed.

"The other pathological condition which favors hæmorrhage, the altered state of the blood, is perhaps more directly influenced by the remedies called styptics. Most of these remedies are astringents, causing contraction of the tonic fibres of vessels and other parts, but some of them also coagulate the blood, and in both these ways they may tend to restrain hæmorrhage."

Concerning the pathological causes of flux and dropsy, the following remarks occur:

"We have thus traced flux and dropsy in common, to elements previously considered, hyperæmia in some of its forms, together with a diseased condition of the blood itself, dependent on defective secretion, or defective nutrition or assimilation. The latter element, although not essential to the production of fluxes or local dropsies, is the chief cause of general dropsy, and constitutes the dropsical diathesis. If we endeavor further to distinguish between the pathological causes of flux and dropsy, we find from observation that flux more commonly results from determination of blood or congestion, with a lax state of the solids, whilst dropsy is rather associated with the altered condition of the blood just noticed."

The subject of inflammation occupies many pages of this work. The manner in which it is treated cannot fail to excite the admiration of the intelligent reader. We earnestly recommend this, as every other part of Dr. Williams' excellent Principles of Pathology, to the diligent perusal of every physician, who is not familiar with the accessions which have been made to medical science within the last few years. Y.

